# Predictive gravity models of livestock mobility in Mauritania: The effects of supply, demand and cultural factors

**DOI:** 10.1371/journal.pone.0199547

**Published:** 2018-07-18

**Authors:** Gaëlle Nicolas, Andrea Apolloni, Caroline Coste, G. R. William Wint, Renaud Lancelot, Marius Gilbert

**Affiliations:** 1 Spatial Epidemiology Lab (SpELL), Université Libre de Bruxelles, Brussels, Belgium; 2 International Center for Agronomic Research and Development, CIRAD, Montpellier, France; 3 Fonds National de la Recherche Scientifique, Brussels, Belgium; 4 Environmental Research Group Oxford (ERGO)—Department of Zoology, University of Oxford, Oxford, United Kingdom; University of Florida, UNITED STATES

## Abstract

Animal movements are typically driven by areas of supply and demand for animal products and by the seasonality of production and demand. As animals can potentially spread infectious diseases, disease prevention can benefit from a better understanding of the factors influencing movements patterns in space and time. In Mauritania, an important cultural event, called the *Tabaski* (Aïd el Kebir) strongly affects timing and structure of movements, and due to the arid and semi-arid climatic conditions, the season can also influence movement patterns. In order to better characterize the animal movements patterns, a survey was carried out in 2014, and those data were analysed here using social network analysis (SNA) metrics and used to train predictive gravity models. More specifically, we aimed to contrast the movements structure by ruminant species, season (*Tabaski* vs. *Non-Tabaski*) and mode of transport (truck vs. foot). The networks differed according to the species, and to the season, with a changed proportion of truck vs. foot movements. The gravity models were able to predict the probability of a movement link between two locations with moderate to good accuracy (AUC ranging from 0.76 to 0.97), according to species, seasons, and mode of transport, but we failed to predict the traded volume of those trade links. The significant predictor variables of a movement link were the human and sheep population at the source and origin, and the distance separating the locations. Though some improvements would be needed to predict traded volumes and better account for the barriers to mobility, the results provide useful predictions to inform epidemiological models in space and time, and, upon external validation, could be useful to predict movements at a larger regional scale.

## Introduction

Many factors that may influence the dynamics and transmission of infectious diseases have been rapidly changing over the last decades. Alongside climate and land use changes, often considered in emerging infectious diseases literature as main drivers, other factors such as the distribution, growth and connectivity of human populations have also been changing rapidly as result of demographic transitions, conflicts or migrations. Similarly, the distribution and connectivity of traded animal populations are strongly influenced by agricultural intensification and changes in trade patterns. The combined effect of these societal and environmental changes taking place simultaneously is difficult to assess, but some, particularly mobility of livestock and traditional trading practices, have been associated with the emergence and the spread of infectious diseases [1–4], and can have strong socio-economic impacts [5,6]. In addition to these long-term trends, culture and tradition strongly shape societies at national, regional and global scale. For example, human population movement during specific periods such as Chinese Spring Festival [7], annual holidays [8], or religious feast around Christmas, Ramadan, Thanksgiving or Hindu Holy feast are known to cause substantially affect global mobility [9] with significant economic and epidemiological implications [10–12]. Large movements of animal populations are also linked to changes in the spatial pattern of food demand, which is anticipated by the market. In low-income countries, such as Sahelian African countries, rapid changes in demand for animal products linked to cultural and traditional events therefore leads to a large number of animals–notably sheep, being slaughtered to meet the seasonal food demand. As a consequence, in the months and weeks preceding such events, trading of live animals is particularly intense. Due to the dry ecoclimate of the Sahelian area, agriculture and breeding strongly depend on the amount of rainfall and the availability of pasture. As a consequence, successive droughts also dramatically affect the Livestock flow. Another example is Madagascar, where kapsile is a traditional practice consisting in an animal barters linked to the labour need in a period when breeders are cash-poor and which strongly affects the patterns of trade flows [3,13]. In West Africa, similar practices, called *loans*, have been described as a large number of short term animal exchanges for reproduction, food supply through milk production, animal traction, *etc* [14]. In Mauritania, the Muslim feast of *Tabaski* or Aïd el-Kebir, is a major cultural and traditional event that strongly influence trading patterns and could have major impact on the spread of diseases. On this occasion, young rams are slaughtered in most families on the 10^th^ day of the month "dhou al-hija", a religious holiday during the last month of the Muslim (lunar) calendar. The date of this festival changes each year according to the Gregorian (solar) calendar and strongly structures the volume of traded sheep during the year. Annual and seasonal differences are thus observed in the sheep trade flows. The country, and the whole Sahel, was hit by severe droughts in the 1960’s, 70’s and 80’s, and more limited droughts later on till 2017 [15]. The drought of 1970 was the main climatic event for the area. Since then, the area has been mostly in deficit of rainfall [16]. The series of droughts had a profound impact on the livestock (affecting 2/3 of the production) and human population. Cattle population dropped, whilst the population of small ruminants increased, the latter being more robust than the former to harsh climatic conditions. Apolloni *et al*. [17] described the livestock trading mobility for the year 2014 in Mauritania, highlighting that the main trading peak related to the *Tabaski* took place between August and December. During this period, the price of male lambs sharply increased, and the high demand strongly affected the trading network structure.

In this paper, we aimed to understand how the *Tabaski* festivity changed the trade network in Mauritania compared to the rest of the year. Apolloni et *al*. [17] provided a comprehensive study of the Mauritania survey data, characterizing the seasonal trade network and ruminant flows within Mauritania, and between Mauritania and the neighboring countries. Here, we first provided a complementary description of inner flows using social network analysis indicators, by contrasting the *Tabaski* and non-*Tabaski* periods, the different ruminant species and modes of transport. Second, we developed predictive models to estimate the probability of a trade connection between two spatial units (areas around markets and/or farms, etc.) based on their potential production and demand characteristics and different measures of the cost distance between them.

## Material and methods

### Study area

Mauritania is situated in the hyper-arid (Sahara) and arid (Sahel) ecozones [18], with low annual rainfall (0–400 mm) concentrated in a short rainy season (June-September). In the northern part of the country, the driest one, only short-cycle plants grow. Livestock, mainly camels and small ruminants, are reared moving around available water points and grazing areas. The southern area, more humid and greener is mostly exploited by transhumant herds. Most of cattle population, being less mobile and demanding more water and nutrients, is concentrated in the southern area, mainly in the region around the river Senegal. Because of the harsh conditions, mobility is a key aspect of animal rearing in Mauritania. Animals are moved almost continuously among grazing areas to optimize the consumption of good quality nutrients. In the absence of slaughterhouses, stocking facilities and road infrastructures, animals are traded alive and butchered at consumption markets.

Past droughts indirectly contributed to the growth of cities, in particular Nouakchott, due to the migration of previous farmers and herders from the countryside to urban areas in search of jobs. Because of this, Nouakchott, the capital city has seen its population dramatically exploding during and following the drought years. As of today, almost one quarter of the total population lives in the capital city (National Bureau of statistics http://www.ons.mr/) Mauritania is still recovering from the latest food crisis in 2011, affecting almost 1 million of its habitants. In 2018, the drought indicators were at the same levels as those of 2012, indicating that the food emergency is not completely over. The inadequate levels of rainfall and the continuous threat of the droughts force herders to sell their livestock, in particular small ruminants, due to shortages of suitable feeding areas.

### Data collection

A survey among veterinary officers was conducted in June 2015 by the National Office for Livestock Research and Development (ONARDEL) to collect their knowledge of ruminant trade flows as reported in Apolloni *et al*. [17]. The survey aimed to monitor the movement patterns during the year 2014. Its results were recorded as a series of trade flow events, with the following information: i) the origin, ii) the destination, iii) the type of trade movement (farm, transit, market), iv) the frequency (annual, monthly, weekly), v) the species (cattle–*Bos indicus*, sheep, goat or camel—*Camelus dromedarius*), vi) the number of heads, vii) the date of the starting of the event, viii) the transportation mode (by truck or on foot) and ix) the latitude/longitude coordinates of the origin and destination. Three types of movements were recorded: i) between pastoral areas for grazing and/or reproduction, ii) from farm to market, iii) or from market to market. Transhumance movements aiming to gradually move herds for suitable pasture areas were not included. The database was cross-checked against sanitary certificates, the scientific documents describing transhumance patterns, and the knowledge of veterinarian staff [17]. Both national and international trade-flows were recorded and the transboundary movements were double-checked through surveys on transit sites between Senegal and Mauritania.

### Additional data

**Fig 1:**
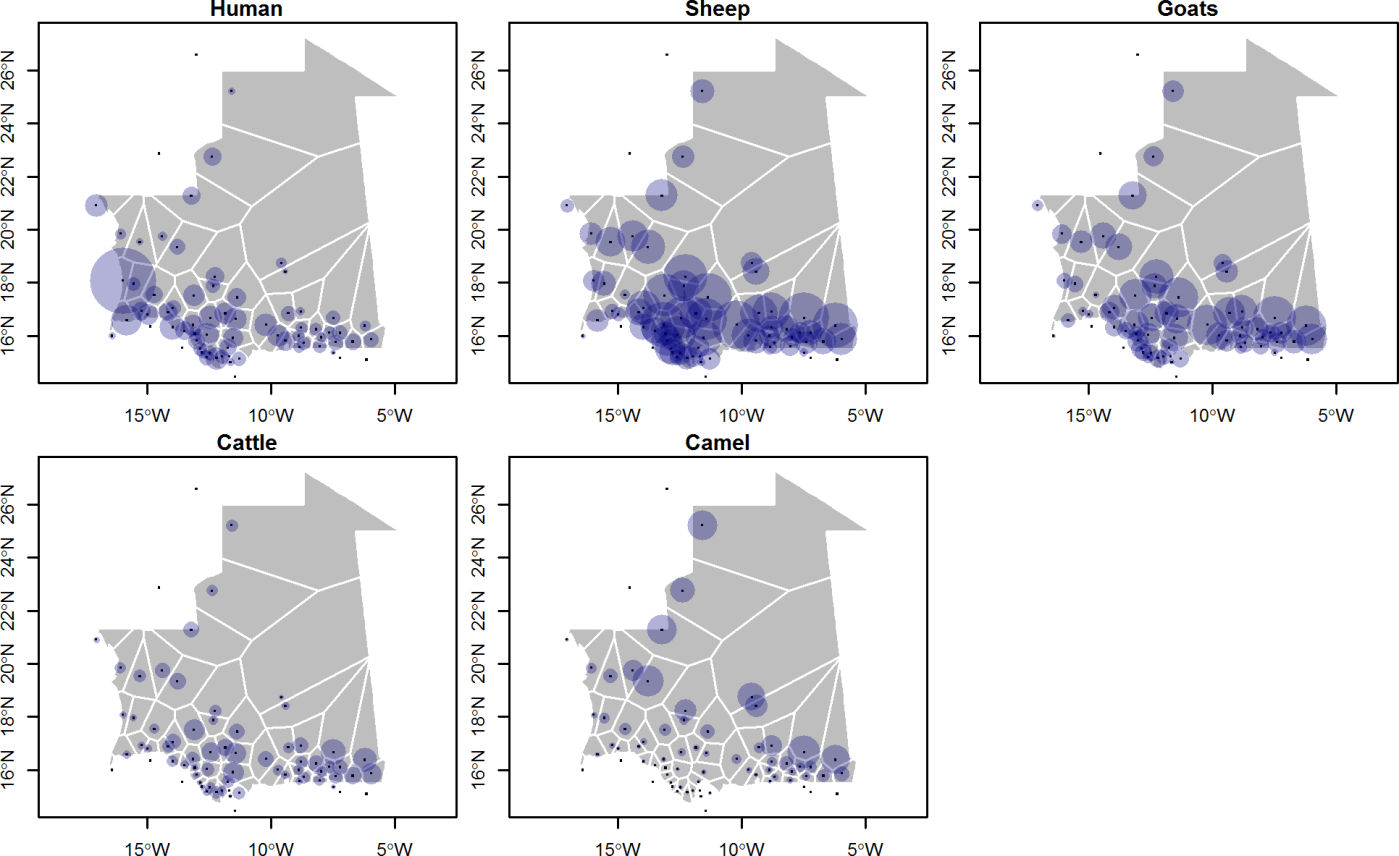
Population of human and ruminants in Mauritania. The size of the circle are proportional to the number of head located within the Voronoï polygon related to the spatial location of the trading network.

In 2013, the Ministry for Rural Development and Environment reported a population of 16.8 million sheep and goats, 1.8 million cattle, and 1.4 million camels for Mauritania to FAOSTAT [19]. Accordingly, and because no finer data was available at national level, we used the most recent version of the Gridded Livestock of the World database (GLW), where the subnational livestock statistics for Mauritania dates back to 2007, and were adjusted to match the FAOSTAT 2010 national totals [20,21]. The WorldPop database was used for the human population [22]. Both databases were aggregated at a spatial resolution of 0.083333 decimal degrees (i.e. approximately 10 km at the equator) (Fig 1). To estimate the cost paths potentially affecting mobility between different localities, we considered two main data sources: the friction layer of Nelson accessibility map, which quantifies the time needed to travel through each pixel [23], and the elevation from the GTOPO30 database (https://lta.cr.usgs.gov/GTOPO30).

### Analysis

In this study, we only considered inner movements of cattle, camels, sheep and goats within Mauritania.

Social network analysis (SNA) [24] have proved to be of significant interest in animals movements analyses in the past decade [25–28]. Here, it was first used to describe the trade networks according to the species, the transport modality (using truck vs. walking), and the season (*Tabaski* vs. non-*Tabaski*). These mentioned periods were defined as strongly influencing the livestock flow within the country by Apolloni *et al*. [17] who investigated the dataset regarding the occurrence of the Muslim festival for the year 2014. A set of network parameters were estimated for the different networks using the simplified definition provided in Wasserman and Faust, (1994) [24]:

- Diameter: a network-level parameter representing the greatest number of links in the shortest path between two nodes.
- Average path length: a network-level parameter measuring the average number of steps along the shortest paths of all possible nodes pairs, i.e. the average number of nodes an actor has to trade through to connect to any other node.
- The clustering coefficient: a node-level parameter of the density of local ties. It measures the probability that neighboring nodes of a node are connected.
- The density: a network-level parameter measuring the proportion of observed links among the possible links between nodes, and indicates how strongly a network is connected.
- Average degree: a network-level parameter quantifying the average number of links connected with a node in a network. Besides these global measures, other centrality measures highlighting the prominent role of nodes in the network were considered: node’s in- and out-degree (the number of connection towards and from each node), node’s in and out-weight (the volume of animal towards and from each node); node’s betweenness (the number of shortest path passing through the node) and node’s eigenvector centrality (scoring the importance of nodes).

Network’s vulnerability to target removal of nodes based on centrality measures and estimates of the size of the largest connected component were tested. The removal of specific nodes, and their links, cause the network to fragment in a set of smaller subnetworks. The size of the largest component can be thought as the maximum extent a disease can spread after the implementation of the control measure (vaccination of animal in the areas surrounding the nodes, market closure, etc.) [29]. Volkova et al. (2010) [30] introduced the notion of epidemic threshold (*q*) in veterinarian epidemiology. This parameter estimates the (minimum) probability for a disease to be transmitted from one node to another to trigger an epidemic. The lowest the epidemic threshold the higher is the risk of an epidemics. This quantity depends on the heterogeneity of the network and the weight’s distribution. In the case of a weighted network the epidemic threshold can be estimated as:

$$q=\frac{\langle w^{out}\rangle }{\langle w^{in}\times w^{out}\rangle }$$

Where 〈 〉 indicates the average value and *w^in^, w^out^* indicate node’s in- and out-weight, respectively. Following the same procedure as in Lancelot et al. (2017) [31], the invasion threshold for each month were estimated. Highlight on the role of occasional links were given (connections appearing just once per year).

We plotted path intersections between different species, transports modalities and seasons, to highlight possible common or specific links for different combinations. In addition, metrics quantifying these intersections were estimated, such as the pairwise percentage of common and specific paths between two networks, respectively.

Gravity models were used to estimate the probability of a link between two distinct nodes according to their features. These models were developed in the field of socio-economics and human migration studies [32,33]. They provide estimates for the flows of goods or people between two nodes, as a function of node-level variables (*e*.*g*. population size, socio-economic factors, *etc*.), and of the distance or movement cost between these nodes. Its most general formulation (Eq 1) shows the analogy with Newton’s gravity law. *MIGij* is the flow between the origin *i* and destination *j*, *p*_*i*_ and *p*_*j*_ are the population at the origin and destination, *d*_ij_ is the distance between *i* and *j*, and *α*, *β* and *γ* are model parameters. The equation is linearized into Eq (2) by a log-transformation, and its coefficients can be estimated with generalized linear models. Eq (2) can be rewritten as a model (3) with an intercept (a flow that would still be present when populations are equal to zero), and a set of predictor variables *x_k_* characterizing the origin or destination, with their associated coefficients *β*_*k*_.

$${MIG}_{ij}=\frac{p_{i}^{\alpha }p_{j}^{\beta }}{d_{ij}^{\gamma }}$$ (1)

$$\log\left(MIG_{ij}\right)=ap_{i}+\beta p_{j}‑\gamma d_{ij}$$ (2)

$$\log\left(MIG_{ij}\right)=\beta_{0}+\beta_{1}p_{i}+\beta_{2}p_{j}+\beta_{3}d_{ij}+\sum_{k=4}^{K}\beta_{k}x_{k}$$ (3)

In this study, we first aimed to estimate the probability of a trade connection between two nodes. Therefore, log(*MIGij*) was replaced with the logit of this probability and logistic regression was used to estimate the coefficients. As the response, all pairs of connected nodes were coded with 1, and all other pairs of nodes were coded with 0. The analysis was split according to the main structuring factors of the networks, i.e. species, transport modality and season. For each sub-model, we tested seven combinations of predictors, considering them both at the origin and destination with inclusion of a distance estimator ((i) great-circle distance, (ii) cost-path distance based on accessibility friction surface or (iii) cost-path distance based on elevation friction surface). The different combinations of predictors that were tested in the models are shown in Table 1. The human and animal populations at the origin and destination were extracted from the Worldpop and GLW raster layers within the Thiessens’ polygons around each node (Fig 1). Thiessen’s polygons represent areas consisting of all points closer to the node than to any other node. These were used because the nodes did not correspond to any particular administrative division that could have been used (i.e. many nodes per admin unit). Although Thiessen’s polygon can produce somewhat misleading long shapes in desertic areas, this is not necessarily a problem as the livestock and human population demographics would be low in these areas anyway. In each sub-model, we first included the extracted animal population of each species at both the source and destination (cattle, sheep, goats, and camels). We used stepwise regression based on Akaike information criterion (AIC) to select a more parsimonious model, with the lowest AIC.

**Table 1:** General equation of the tested models.

model	general equation
***E1***	H*pi* + H*pj* + *d*_*ij*_g
***E2***	L*pi* + L*pj* + *d*_*ij*_g
***E3***	H*pi* + H*pj* + L*pi* + L*pj* + *d*_*ij*_g
***E4***	H*pi* + H*pj* + L*pi* + L*pj* + *d*_*ij*_a
***E5***	H*pi* + H*pj* + L*pi* + L*pj* + *d*_*ij*_el
***E6***	Δ(H*pi*, H*pj*) + Δ(L*pi*, L*pj*) + *d*_*ij*_g
***E7***	E3 + mode + mode: *pi* + mode: *pj* + mode: *d*_*ij*_g

***pi*:** *population at the origin (L*: *livestock*, *H*: *human);* ***pj*:** population at destination *(L*: *livestock*, *H*: *human)*; ***d***_***ij***_: distance (g: great circle distance, a: costhpath distance based on accessibility friction surface, el: costhpath distance based on elevation friction surface), mode: transport modality (by truck or on foot).

In a second step, this analysis was repeated using the number of animals traded between these two locations as the response variable. To better differentiate the factors influencing the trade probability from those influencing its volume, the latter analysis was restricted to pairs of locations with an existing trade link. All analyses were coded and carried out using R [34]. The “sna” package was used to describe and analyse the trade network [35].

## Results

**Fig 2:**
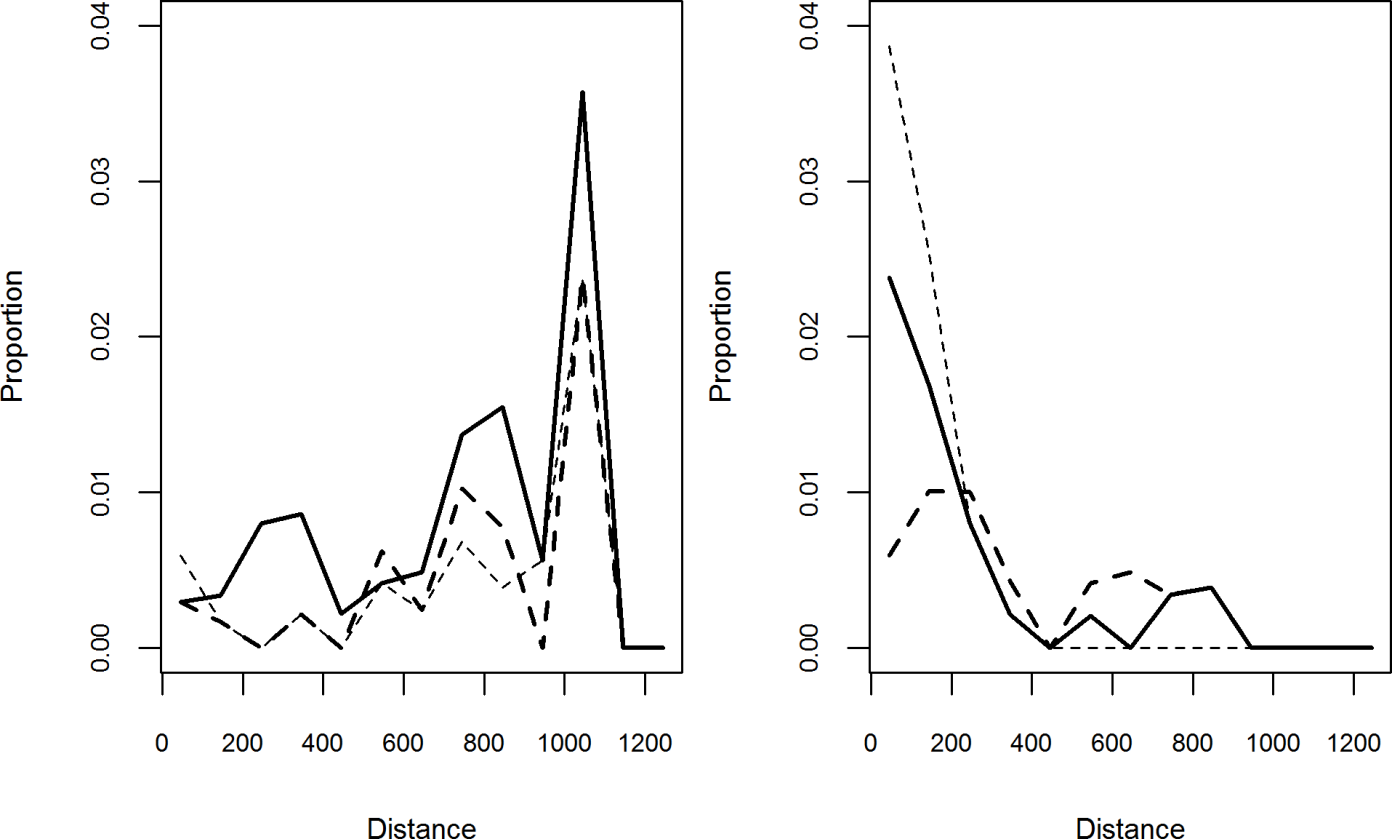
**Great-circle distance of the national movements by truck (left) and on foot (right) recorded in the survey.** Distance are given in kilometers for each species (thick line: small ruminants, dashed line: cattle, thick dash line: camel).

The dataset consisted in 2,219 trade movements involving 7.1 million head. The subset of national movements, the focus of this analysis, included 1,178 movement events involving 2.1 million head (Table 2). International movements involved around 5 million head, sold or bought to or from Senegal, Mali, Ivory Coast, Guinea Bissau and Morocco. As the destination of these international movements within these countries was unknown, they could not be included in the network analysis or gravity models. Within Mauritania, the trading network was composed of 65 nodes and 84 unique paths. Transport by foot was the most represented mode, corresponding to 83% of the animal flow (Tables 2 and 3). The foot and truck transport modalities presented contrasted patterns. The largest share of foot movements being short to medium distance (0–200 km), whereas the opposite was observed for movements by truck, where the largest share was represented by movements > 500 km (Fig 2). Similarly, those contrasting patterns somewhat matched the seasonal pattern. The *Tabaski* period represented 41% of the inner animal movements, with 57% of them by truck. In contrast, only 25% of the head were moved by truck outside the *Tabaski* period (Table 2).

**Table 2:** Number of national and international traded animals in Mauritania. Values represent the number of animals moved for each given period. Movements are defined as national if both the origin and destination are located within the Mauritanian border. Small ruminants (SR) include sheep and goats. Some of the records did not differentiate the species individually and are counted as “Cattle and SR” or “Mixed”.

	Cattle	Cattle and SR	Camels	SR	Mixed	Total
Truck	67 240		43 760	1 105 870		1 216 870
**Aug-Dec**	**34 398**		**19 119**	**843 047**		**896 565**
international	2 400		2 120	410 500		415 020
national	31 998		16 999	432 547		481 545
**Jan-July**	**32 842**		**24 641**	**262 822**		**320 305**
international	9 600		3 280	980		13 860
national	23 242		21 361	261 842		306 445
**Foot**	**1 801 846**	**1 660**	**598 034**	**3 334 755**	**96 563**	**5 832 859**
**Aug-Dec**	**342 444**	**277**	**112 740**	**716 645**	**19 740**	**1 191 847**
international	283 403		28 011	518 420		829 835
national	59 041	277	84 729	198 225	19 740	362 012
**Jan-July**	**1 459 402**	**1 383**	**485 294**	**2 618 110**	**76 823**	**4 641 012**
international	119 1123		271 755	2 286 295		3 749 172
national	268 279	1 383	213 539	331 815	76 823	891 839
**Total**	**1 869 086**	**1 660**	**641 794**	**4 440 625**	**96 563**	**7 049 729**

**Table 3:** Network parameters and number of links of the national movement networks in Mauritania. Links are provided according to the transport modality and *Tabaski* periods (*Tabaski*: Aug–Dec; Non-*Tabaski*: Jan–July).

		All species	Cattle	SR	Sheep	Goat	Camel
**Network-level parameter**	*Number of nodes*	65	65	65	65	65	65
	*Number of links*	84	49	56	54	32	34
	*Diameter*	5	4	5	5	3	2
	*Clustering coefficient*	0.19	0.15	0.18	0.18	0.15	0.03
	*Average path length*	1.84	1.56	1.73	1.73	1.34	1.13
	*Density*	0.020	0.012	0.013	0.013	0.0077	0.0082
	*Average degree*	2.65	1.51	1.72	1.66	0.985	1.05
**Number of link**	*Transport modality*						
	*Truck*	*33*	13	28	28	13	14
	*Foot*	*55*	37	30	28	20	22
	Intersection*	*2*	2	2	2	1	2
	*Trading period*						
	*Non-Tabaski*	*73*	45	46	44	29	31
	*Tabaski*	*57*	31	39	39	19	21
	Intersection*	*44*	27	14	29	16	18

*Intersection: number of link which are present in both networks. Truck/Foot: respectively, links which involved movement of animals by truck or on foot; SR: Small ruminants.

**Fig 3:**
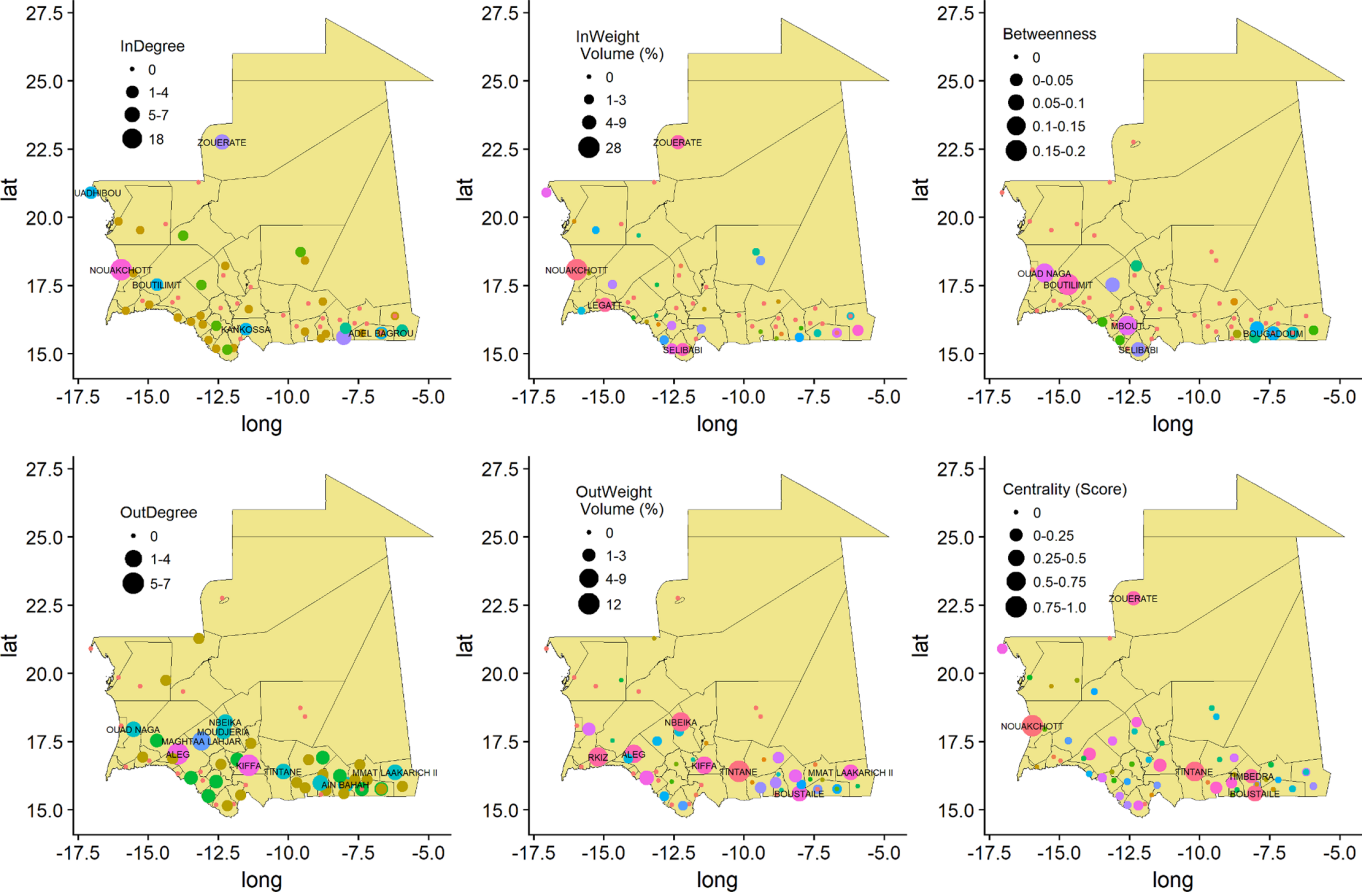
Network’s centrality measures of the Mauritanian’s livestock trade. Node size show the importance of the measured centrality values. in and out-weight measures were scaled on the total volume of traded livestock; eigenvector centrality (centrality measure) were scored from 0 to 1, betweenness was considered for the fraction of paths passing through the node.

The Mauritanian network was weakly connected. The density value indicated that 2% of possible node pairs were actually connected, and the network level centrality parameters and clustering coefficient were low (Table 3). However, each of the exchange networks (full network, or species-specific sub-networks) contained a single component in which the average length of the shortest path between node pairs was lower than 2 links, the maximum value (diameter) being 5 links. The goat and camel trading networks were smaller with a diameter of 2 and 3 links. On average, in the full species network, a given node was directly connected with approximately 2 other nodes on average (average degree). Both the in-degree and in-weight distributions are right-skewed. Only 5 locations from the livestock trading network attracted more than 50% of the connections, and are also the destination for more than 60% of the traded volume. Nouakchott, the main urban consumption market of the country, acts as hub for livestock mobility with 18 links which concentrate around 1/3 of the traded animals’ total volume (Fig 3, Table 4). With the highest centrality score, the capital city is the most important node of the network. Almost 2/3 of the nodes has at most one outgoing link, whilst Aleg and Kiffa, in the southern region, are connected to other 7 localities, and Tintane has the largest out-weight. Finally, the betweenness distribution is right-skewed and Boutilimit appears to be the node with highest betweenness. Few of the centrality measures are significantly correlated (p-value<0.05) and reported in Table 5.

**Table 4:** List of nodes with highest values for centrality measures. Each column corresponds to a specific network (all species or by single species) Each line relates to a specific centrality measure. Only the name of the node corresponding with the largest value for each measure is reported. In the case of multiple nodes with same value of the measure, all the names are reported.

	All species	Cattle	SR	Camel
***Indegree***	Nouakchott	Nouakchott	Nouakchott	Nouakchott
***Inweight***	Nouakchott	Selibabi	Nouakchott	*Zouerate*
***Outdegree***	Aleg	Aleg Kiffa	*Aleg*	*Kiffa*
	Kiffa	Mmat Laakarich II		*M*. *Lahjar*
***Outweight***	Tintane	Kaedi	Tintane	*Nbeika*
***Betweenness***	Boutilimit	Adel-Bagrou	Boutilimit	Boutilimit
		Mbout		Nbeika
***Eigenvector Centrality***	Nouakchott	Kaedi	Nouakchott	Nbeika

**Table 5:** Correlation coefficients among centrality measures. Pearson correlations coefficients among centrality measure. Only significant (p-value <0.05) coefficients are reported.

	*Indegree*	*Outdegree*	*Inweight*	*Outweight*
***Indegree***			0.88	
***Outdegree***				0.63
***Inweight***	0.88			
***Outweight***		0.63		

**Fig 4:**
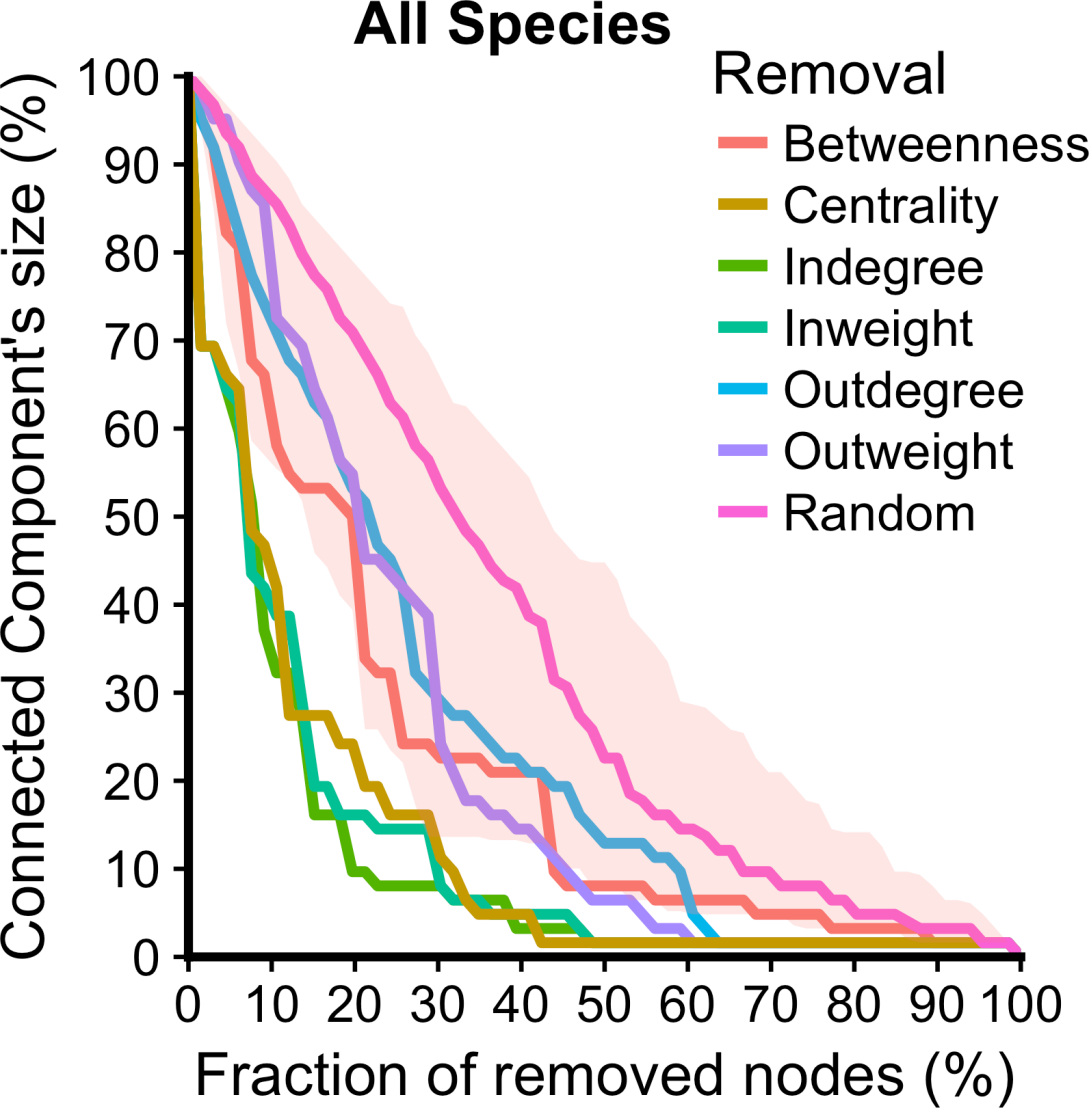
Effect of targeted removal on the connected component size. Values on the x axis indicate the percentage of nodes removed (cumulative), together with their links. Values on y axis indicate the percentage of nodes in the largest connected sub-network, after the removal. Color indicates the removal procedure based on centrality measure score (starting from the highest score nodes) or randomly. Shaded areas correspond to 95% CI for the random procedure.

Results of the percolation analysis on networks cohesion are shown in Fig 4. Nodes are removed based on the centrality measures (indegree, outdegree, incoming and outgoing volume, betweenness, eigenvector centrality) and, for comparison purposes, randomly. Removing nodes in order of their incoming connections, incoming volume and centrality result as the most effective strategies of fragmentation. In the first case, removing less than 20% of the nodes (13 nodes) results in decomposing network in a set of subnetworks whom the largest one contains less than 10% of the nodes (7 nodes).

**Fig 5:**
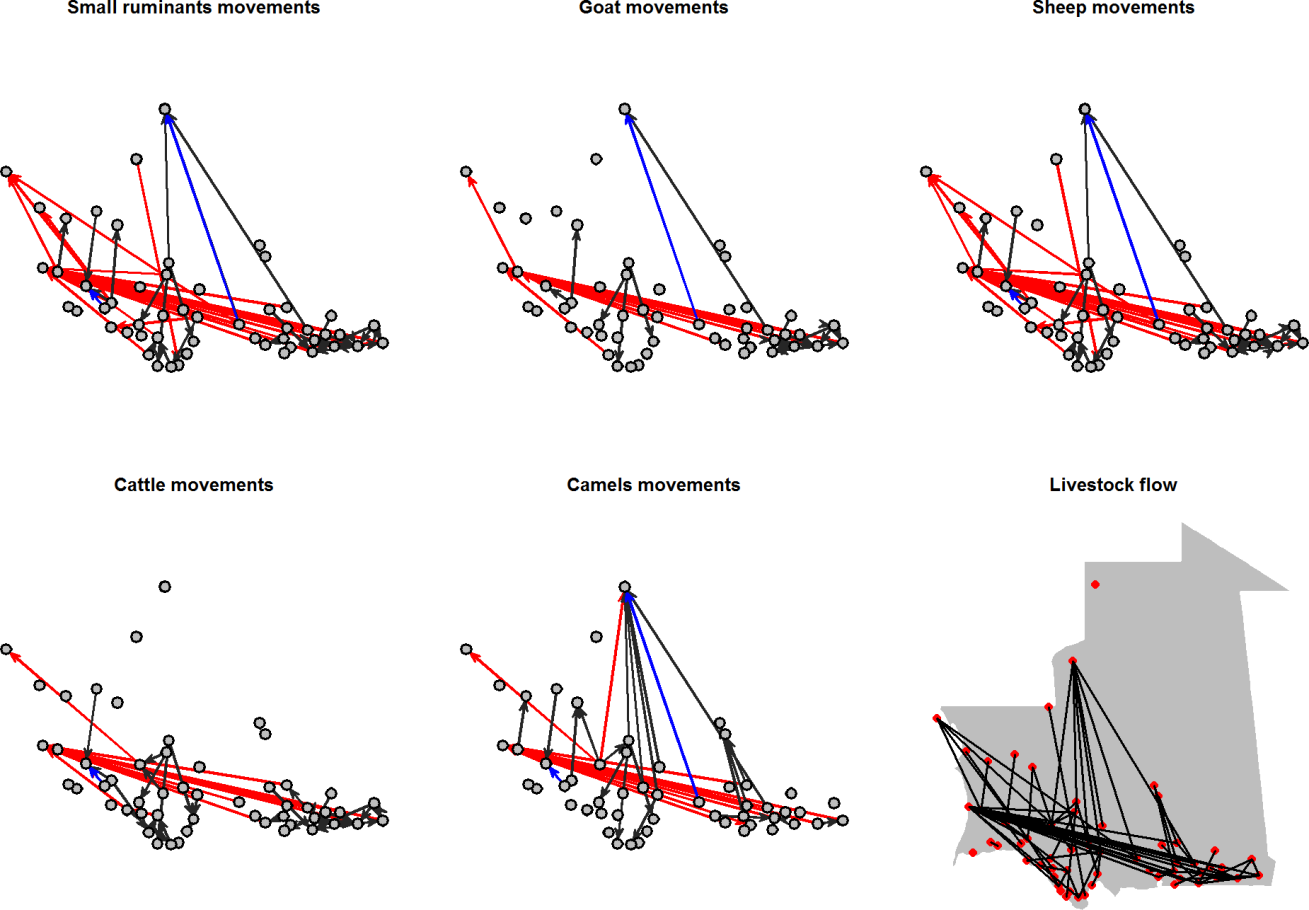
Species-level movement networks. Each diagram represents the movement links that are specific to the species (red: by truck, dark grey: by foot, both truck and foot: blue). The last plot shows the entire network of all species.

In total, 94% of the goats trading network paths were shared with the sheep (Fig 5, Table 6). These two networks were merged into a single small ruminant trading network in the gravity models. A high degree of overlap between pairs of species networks was highlighted (Table 6). However, overlapping only involved 20% of the full set of trading links (Fig 5, Table 6). Among the remaining links, many were species-specific: 30% of the small ruminant (17/56), 33% of the cattle (16/49) and 32% of the camel (11/34) links were not included in any of the other networks. Very few links were shared between the truck and foot networks and were identical for all species (2/84, Table 6).

**Table 6:** Comparison of the trading networks of the different species. The values correspond to the percentage (*italic*) and the number (bracket) of common links and difference between the trading network of the pair of species.

	network species 1	network species 2	Intersection % (n)	Difference Sp1/Sp2 % (n)	DifferenceSp2/Sp1 % (n)
***Cattle/SR***	49	56	*65* (32)	*35* (17)	*43* (24)
***Camel/SR***	34	56	*65* (22)	*35* (12)	*61* (34)
***Camel/Cattle***	34	49	*47* (16)	*53* (18)	*67* (33)
***Goat/Sheep***	32	54	*94* (30)	*6* (2)	*44* (24)
***All species*** *	84	-	*20* (17)		

* All species: Association of small ruminants, Cattle and Camel trading networks.

The role of nodes could change depending on the species considered. Previous results about most central nodes hold when we consider the “small ruminants only” network. Whilst Nouakchott remains always the most connected node, most of the volume of cattle and camels are directed towards Selibabi and Zouerate, respectively. The markets of Kaedi, for cattle, and Nbeika, for camels, become more central in the network (see S1 File and Table 4). In term of cohesiveness, specie-specific network are more vulnerable to the target removal of nodes. As in the all species case target removal based on indegree and inweight are the most efficient procedures. In fact, in both cases, removing a limited quantity of nodes, 8 for camel’s network 10 for cattle’s one and 12 for small ruminants one, results in decomposing the respective networks in a set of subnetworks whom the largest one contains at most 8 nodes. This is particularly relevant for small ruminants and the cattle networks, whose largest subnetworks consist of 7 and 10 nodes, respectively, after the removal of only 3 most connected nodes.

**Fig 6:**
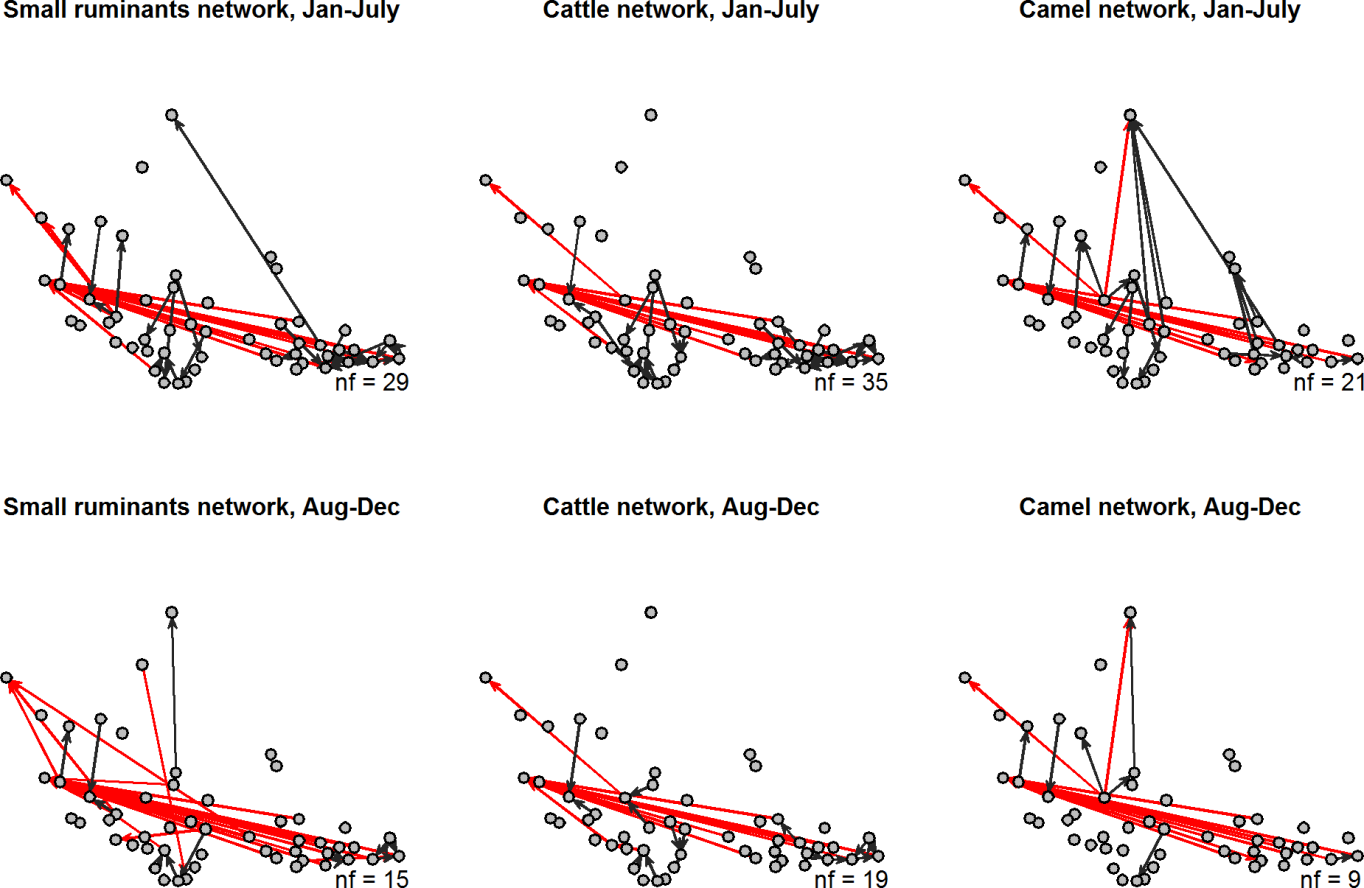
Movement networks according to the species and seasons. Note that for each season the links present in both defined transportation mode are not plotted and are the same for both defined season (red: by truck, dark grey: by foot, nf: number of movement links by foot, Jan-July: Non-*Tabaski* period; Aug-Dec: *Tabaski* period). Fig 3 should be considered to see the redundancy between transportation mode (blue edge).

There was a high number of shared links between the *Tabaski* and non-*Tabaski* periods (44/84, Table 6). Both *Tabaski* and non-*Tabaski* networks included truck and foot movements, but their relative proportion was different. During the *Tabaski* period, there were fewer foot movements and higher truck movements (Fig 6). Very few trade connections involved both truck and foot movements in the two periods (Table 6, Figs 5 and 6).

**Fig 7:**
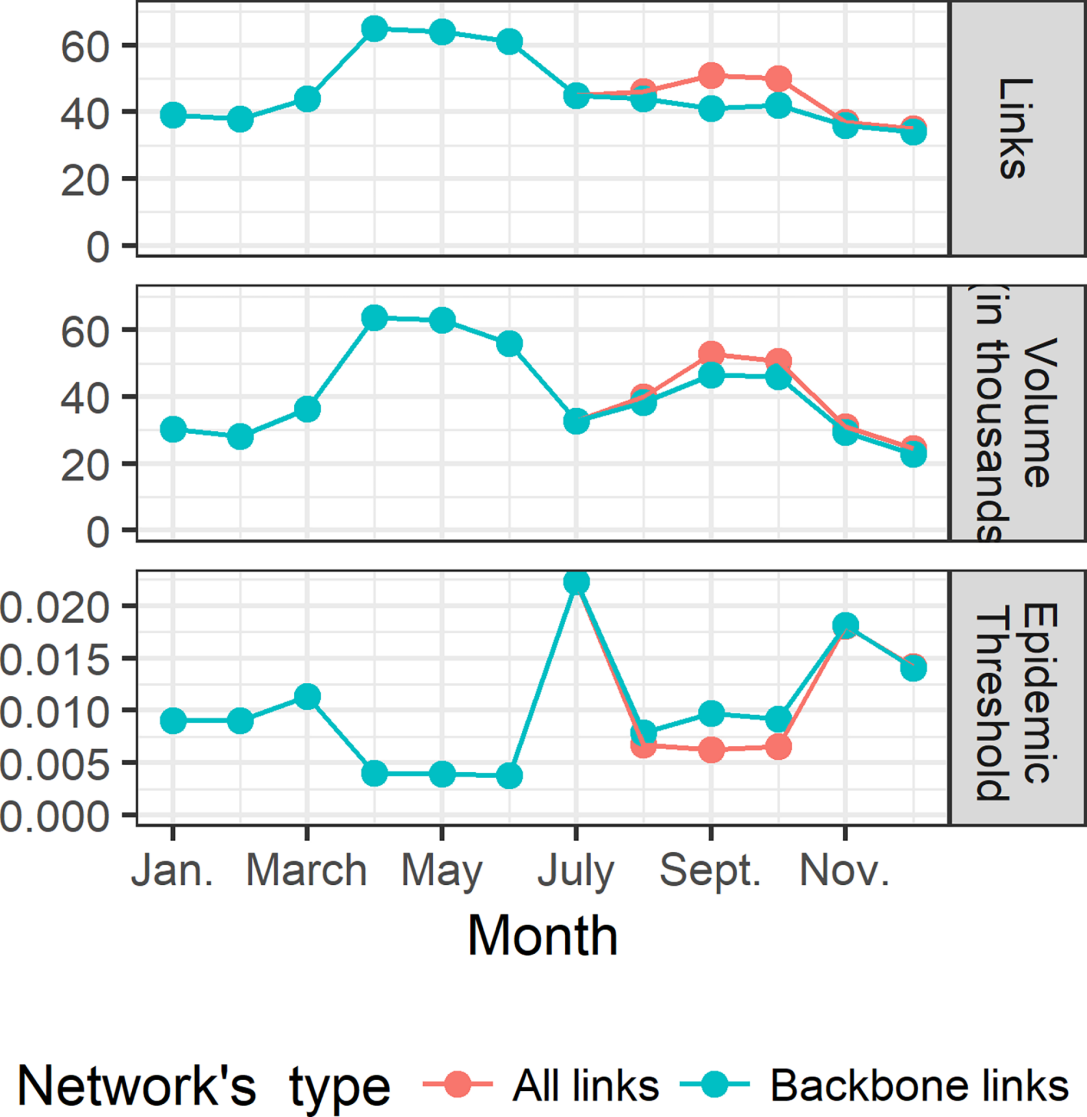
Variations of network quantities along the year. Bottom: monthly epidemic threshold variations along the year; Centre Volume of animals traded during the month; Top number of links active. For each month, we have considered 2 networks: all network including occasional links appearing on that month; backbone, excluding occasional link. The different colors correspond to the quantity evaluated for the specific network.

As the network changes along the year so its proneness to diffuse diseases. Fig 7, present the variation of the epidemic threshold (denoted *q*), estimated every month, along the year in comparison with the number of links active and the volume of livestock traded. Since the network can change along the year, particularly new active links around *Tabaski*, we considered the backbone network from Apolloni et *al*. [17] (containing links present more than 2 months) and the total network containing all the link active that month to elicit the role that *Tabaski* plays on the risk of transmission. The epidemic threshold is at the lowest values between the March and June, when the volume and the number of exchanges (links) is at maximum, and around *Tabaski*, when a second peak of movements (mainly small ruminants) whose volume is almost equal to the first peak, is observed. Occasional links, appearing only for the *Tabaski* reason, decreases the invasion threshold and consequently the risk of disease spreading is higher in this period.

Table 7 presents the different sets of gravity models that were applied to the full network (binary outcome: 1 if two nodes were connected and 0 otherwise), and to the networks broken down by species, period and transport modality.

**Table 7:** AIC values of the logistic regression models. The models are broken down by species (SR: small ruminants, CT: cattle, CM: camels), period (Tab: *Tabaski*, NTab: Non-*Tabaski*) and transport modality. E1-E6 correspond to different models described in Table 1.

AIC	model	n link	n unique link	E1	E2	E3	E4	E5	E6	E7
ALL SPECIES	All	1178	140	1501.8	1604.1	1464.1	1488.9	1485.1	1461.9	1342.7
	NTab	518	53	1339.8	1418.6	1303.3	1323.7	1323.6	1303.3	
	Tab	660	87	995.6	1133.7	979.8	983.1	982.9	976.1	
	Truck	735	121	427.2	601.1	405.0	408.9	407.3	416.9	
	Truck—NTab	443	91	315.7	470.7	302.4	309.1	305.4	307.8	
	Truck—Tab	257	40	175.2	280.5	172.0	174.9	172.2	175.2	
	Foot	129	21	899.3	885.9	877.5	951.1	934.7	885.6	
	Foot—NTab	478	81	843.8	832.2	821.8	886.8	874.0	832.1	
	Foot—Tab	182	41	460.4	454.7	455.2	498.3	484.9	455.7	
SR	All	620	58	589.5	639.7	572.0	578.9	576.7	570.3	
	NTab	374	46	482.7	530.3	470.1	474.1	476.5	470.7	
	Tab	246	40	412.9	473.1	404.0	404.2	403.7	403.7	
	Truck	324	31	210.3	290.5	204.5	205.7	205.7	205.9	
	Foot	296	27	315.3	308.2	304.7	331.3	327.0	307.9	
CT	All	341	47	494.1	526.9	480.6	474.9	487.2	489.4	
	NTab	214	43	465.0	492.4	451.3	444.5	455.8	459.3	
	Tab	127	31	342.3	384.2	329.3	322.5	328.0	340.2	
	Truck	109	13	107.2	146.7	105.6	105.9	106.0	106.0	
	Foot	232	34	305.7	295.8	296.0	304.1	307.9	307.7	
CM	All	217	35	411.5	425.1	399.2	397.4	399.5	395.8	
	NTab	147	32	390	393.9	377.0	375.9	377.6	373.4	
	Tab	70	20	241.7	265.8	236.1	235.7	236.1	233.4	
	Truck	113	13	116.6	156.3	109.9	110.8	108.9	112.3	
	Foot	104	22	266.6	258.7	261.3	264.9	265.9	258.6	

The best results were obtained with models including human and animal populations at the origin and destination, with a great-circle distance (model E3, Tables 1 and 7). Replacing this distance with cost-distance functions of accessibility (E4) or elevation (E5) did not improve the results. Similarly, although we noted some improvements for some combinations of species and transport modalities, the use of the population difference between origin and destination rather than their absolute values (E6) did not lead to improved models for the different breakdowns. In all models, and for all species networks, when the animal population was kept as a predictor, the number of sheep was the most important predictor.

**Fig 8:**
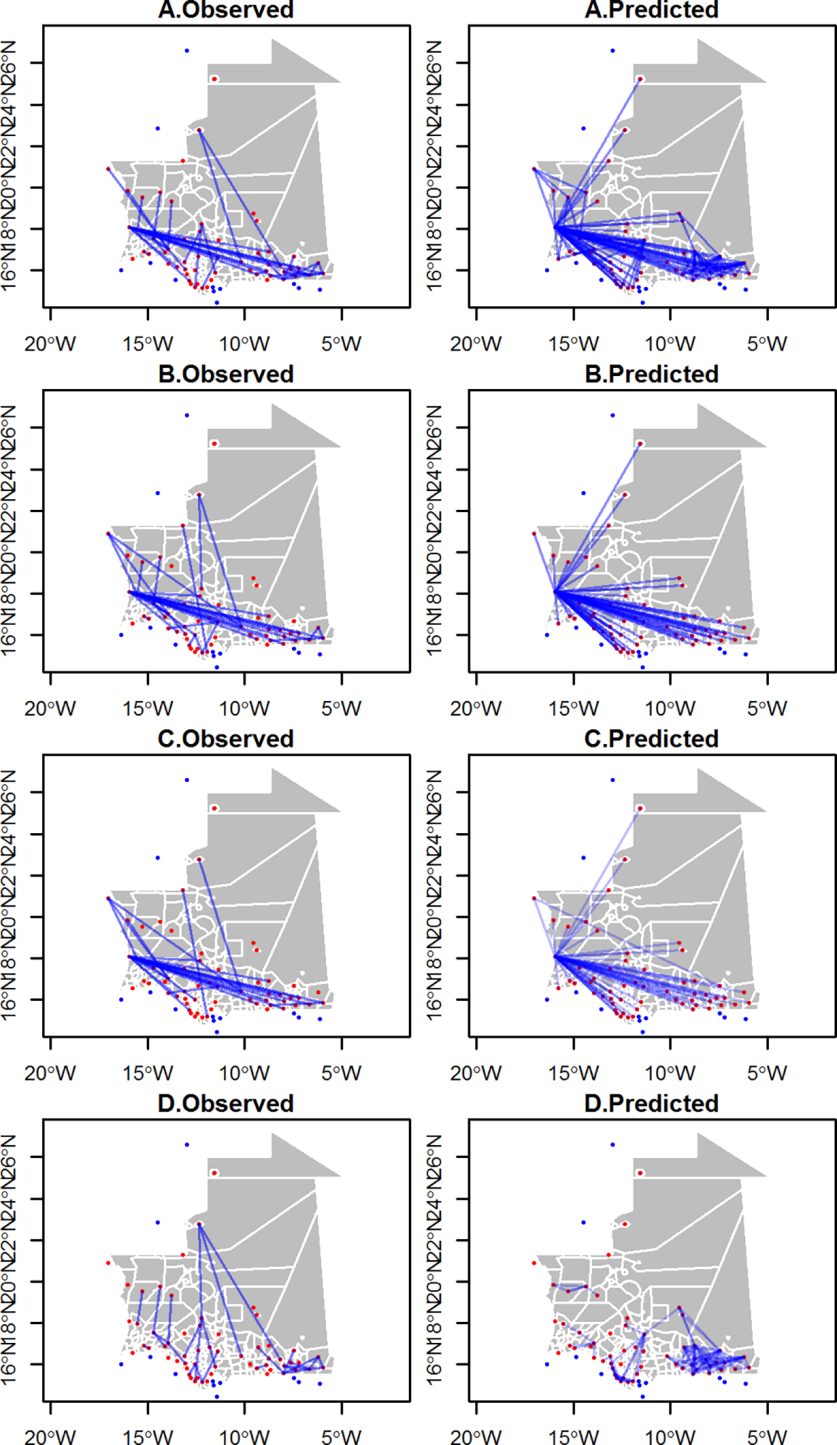
Observed and predicted movements links from the gravity model applied to the small ruminant networks. Models were applied to movements occurring within the non-*Tabaski* period (A), the *Tabaski* period (B), without distinction of period (C-D): movements by truck (C) and by foot (D).

Table 8 presents the details of the final models broken down by species, season and transport mode with the human and sheep population at the origin and destination and great-circle distance as predictors. The predictive power of the models were moderate to very good, according to the species, season and mode of transport, with AUC values ranging from 0.76 to 0.97. Considering all species, seasons and transport modes, positive associations were found between the probability of a trade connection and: i) low human population at the origin, ii) high human population at the destination, iii) high sheep population at the origin, iv) low sheep population at the destination, and v) a low great-circle distance (Table 8). The results were similar for the small ruminants’ network model, whereas for the cattle model, the human population at the origin and the sheep population at the destination were not significant. For the camel network, the human population at the origin and the great-circle distance were not significant. The sign of the significant effects was coherent across the sub-models, i.e. a higher probability of trade event was always associated with low human population at the origin, or high human population at the destination, a high population of sheep at the origin, a low population of sheep at the destination and a low great-circle distance, or a combination of these effects. In addition, meaningful differences were noticed in some sub-models. For example, in small ruminant models split by transport mode, human population at the destination, and sheep population at the origin were not significant in the foot-movement sub-model. In contrast, both predictors at the origin were not significant in the truck-movement sub-model. Great-circle distance and both human and sheep population were not significant for the latter, though. The observed and estimated trade links are illustrated in Fig 8 for these small ruminant models for the *Tabaski* and non-*Tabaski* periods, and movements by trucks or on foot. During the non-*Tabaski* period, the fitted values correctly captured the co-existence of short- and long-distance movements, whereas long-distance movements were prominent during the *Tabaski* period. Similarly, the prediction of the truck or foot movements corresponded to their respective long or short distances. None of the models correctly fitted the south/north movements.

**Table 8:** Multivariate linear model and significance of the parameters given for the selected model (E3).

		Intercept	Hpop_i_	Hpop_j_	Spop_i_	Spop_j_	Great-circle distance	AUC
*All mode*	*All species*	-4.239 ***	-7.622 10^−6^ *	4.667 10^−6^ ***	3.905 10^−6^ ***	-5.104 10^−6^ ***	-2.278 10^−3^ ***	0.836
	*Small ruminants*	-3.895 ***	-1.0 10^−5^	4.92 10^−6^ ***	2.45 10^−6^ ***	-4.00 10^−6^ **	-2.129 10^−3^ ***	0.840
	*Cattle*	-4.619 ***	-8.928 10^−6^	5.596 10^−6^ ***	2.118 10^−5^ ***	7.090 10^−6^	-4.416 10^−3^ ***	0.884
	*Camel*	-5.402 ***	-1.966 10^−6^	4.189 10^−6^ ***	4.699 10^−6^ **	-6.374 10^−6^ *	-5.632 10^−4^	0.790
*All non-Tabaski*	*All species*	-4.210 ***	-1.069 10^−5^ **	4.555 10^−6^ ***	4.196 10^−6^ ***	-5.356 10^−3^ ***	-2.415 10^−3^ ***	0.840
	*Small ruminants*	-3.809 ***	-1.751 10^−5^ **	5.08 10^−6^ ***	2.634 10^−6^ **	-3.575 10^−6^ *	-2.628 10^−3^ ***	0.863
	*Cattle*	-4.599 ***	-1.239 10^−5^ *	5.417 10^−6^ ***	2.244 10^−5^ ***	4.982 10^−6^	-4.095 10^−3^ ***	0.885
	*Camel*	-5.563 ***	-1.559 10^−6^	3.852 10^−6^ ***	5.167 10^−6^ ***	-6.139 10^−6^ *	-5.802 10^−4^	0.781
*All Tabaski*	*All species*	-5.355 ***	-3.679 10^−6^	5.424 10^−6^ ***	3.563 10^−6^ ***	-4.107 10^−6^ **	-1.595 10^−3^ ***	0.869
	*Small ruminants*	-4.680 ***	-5.409 10^−6^	5.408 10^−6^ ***	1.985 10^−6^ *	-4.637 10^−6^ *	-1.378 10^−3^ *	0.863
	*Cattle*	-6.179 ***	-3.851 10^−6^	6.665 10^−6^ ***	2.269 10^−5^ ***	1.598 10^−5^ *	-3.287 10^−3^ ***	0.901
	*Camel*	-6.391 ***	-4.458 10^−7^	5.102 10^−6^ ***	4.862 10^−6^ *	-7.167 10^−6^	-2.454 10^−4^	0.837
*Truck All period*	*All species*	-5.841 ***	-7.730 10^−8^	6.876 10^−6^ ***	3.242 10^−6^ *	-2.072 10^−5^ ***	1.126 10^−3^ *	0.934
	*Small ruminants*	-5.432 ***	-9.748 10^−7^	6.600 10^−6^ ***	1.634 10^−6^	-8.942 10^−6^ *	8.968 10^−4^	0.91
	*Cattle*	-2.574 ***	-1.350 10^−7^	3.652 10^−5^	1.903 10^−5^	9.883 10^−6^	-1.049 10^−2^ ***	0.945
	*Camel*	-6.015 ***	8.635 10^−7^	7.230 10^−6^ ***	4.261 10^−6^	-3.349 10^−5^ *	1.590 10^−3^	0.966
*Foot All period*	*All species*	-2.665 ***	-1.396 10^−5^ **	-1.435 10^−6^	4.702 10^−6^ ***	-3.418 10^−6^ **	-5.783 10^−3^ ***	0.830
	*Small ruminants*	-2.225 ***	-1.963 10^−5^ *	3.289 10^−6^	3.289 10^−6^	-2.996 10^−6^ *	-6.409 10^−3^ ***	0.853
	*Cattle*	-6.470 ***	9.398 10^−7^	6.589 10^−6^ ***	1.636 10^−5^	-1.105 10^−4^	8.720 10^−4^	0.908
	*Camel*	-4.200 ***	-3.373 10^−6^	-4.043 10^−6^	-4.940 10^−6^ **	-4.366 10^−6^	-1.982 10^−3^	0.759

*** p<0.001

** p<0.01

* p<0.05

NS: not significant

Hpop: Human population at the origin (i) and at destination (j); Spop: Sheep population at the origin (i) and at destination (j).

None of the gravity models with the flow of animals as the response, and the same set of predictors as in the presence/absence model detailed above, showed any significant association.

## Discussion

Though international movements were not addressed in this study, it is noteworthy that in 2014, the largest sheep exportation peak was observed during the “soudure”, i.e. the period separating the end of familial cereal reserve saved after the previous harvest (millet, sorghum…) from the next harvest. This was also the hot, dry season, when forage and surface water resources were finished, and ruminant livestock starved. Therefore, the most obvious option for the Mauritanian livestock farmers was to sell most of the offspring, only keeping the core of reproductive ewes and she-goats. In addition, many of the latter spent this season in the closest areas with more abundant pastoral resources, i.e. in Senegal and Mali. Short- and mid-distance movements–most of them by foot, thus allowed pastoralists to exploit more suitable environmental conditions and reduce the economic cost of feeding the animals. In future years, when the *Tabaski* feast occurs during the soudure period, the relative importance of truck vs. foot movements may change and add to each other. Moreover, as the *Tabaski* is a mobile festivity which is celebrated annually among Muslims worldwide 70 days after Ramadan, a time slip of 10 days upstream occurs each year. An overlap with specific diseases vectors may thus occurs depending on the occurrence of the feast (year-dependence of the epidemic risk). Around Senegal river, *Aedes* mosquitoes show a peak in the periods of July-August and September during the rainy season. Thanks to the happening of the *Tabaski*, held in the last years between September and October, the risk of infection was elevated [36]. The risk remains high every year due to the large volume of moved animals and the traders’ preference for truck transportation and its fastness which allow viremic animal introduction on remote locations.

The low values for the national network diameter, the presence of hubs and the low values of the epidemic threshold, indicate that the network could be prone to transmission of diseases. This means that also a lowly transmitted disease, once introduced in the national network, could reach all nodes (the network’s single component) in a short amount of time (small diameter). On the other hand, independently of the species considered, the mobility network is prone to fragmentation due to targeted intervention based on nodes activity (in-degree and in-weight). After the intervention, the network is decomposed in a set of smaller subnetworks, and virus can circulate only among nodes of the same sub-network. Vaccinating animals in largest markets (nodes with largest number of incoming animals) or closing these markets, could result in a very effective way of controlling the epidemic spread.

Regarding internal movements, the gravity models correctly predicted the probability of a trade connection and their interpretation was straightforward. Locations with few sheep and high human populations, i.e. urban consumption centers, acted as movement sinks. Conversely, areas with high sheep and low human populations, i.e. rural livestock farming areas, acted as movement sources. For a similar level of production and demand, short distance movements were more likely that long distance ones. In addition, Nouakchott, the capital city located on the coast, strongly influenced the network structures. Almost a quarter of the total population of Mauritania lives in Nouakchott (according to the National Statistical Office http://www.ons.mr/) and because of this, it is the largest terminal market with an incoming volume of almost 30% of the national one. In preparation for *Tabaski*, almost 50% of the total traded small ruminants are sold in Nouakchott. The largest majority of livestock provisioning Nouakchott markets comes from the South-Western area of Mauritania. A continuous flux of animals is ensured by stockists who collect animals at collection market and transfer them by truck to the capital city. Here, stockists buy imported goods, arrived at the international port, to sell at collection markets.

It might look odd that the sheep population at the origin and destination of movements was the best predictor for all species-level networks. A possible explanation might be that the spatial distributions of all ruminant species were positively correlated, thus making the sheep population a confounding factor for the other ruminant populations. This assumption was corroborated by the dominance of small ruminant movements: 1.3 million vs. 0.39 million for cattle and 0.37 million for camels. Therefore, the small ruminant network probably influenced all other ruminant trade activities.

The seasonal models had good predictive power for the models of both truck or foot movements. These models captured the dominance of long-distance movements (truck movement network), of short-distance movements (foot movement network) and different combinations of short and long-distance movements (*Tabaski* vs non-*Tabaski* periods). However, the models did not capture some of the trade links. For example, they failed to predict the link to the northern city of Zouerate, which was involved in the small ruminant and camel trading networks. Located in the desert and with a low accessibility, this city has a large iron mining industry, and virtually no local production. The working population there may be much higher than the population estimates in the Worldpop population database, which maps people according to their residence, not their working place. Such high populations of workers may generate high demand for small ruminant and camel meat, and camel milk. Armed conflicts may also influence the pattern of demand with drivers not accounted for in the models. For example, an important flow of human population was reported in south-eastern Mauritania coming from Mali after the terrorist attack of January 2013. These refugees established camps close to the border with Mali: their population may have influenced the pattern of demand and production in a way that was not captured by the model.

The use of cost-distance instead of great-circle distance did not improve the models. The cost-distance was estimated using the accessibility friction map which is based on the road network and land-use data, two expected drivers of the livestock trade networks. However, the friction layer might not be adapted to the specific constrains of animal movements in arid and hyper-arid environments. For example, the presence of water points or stopover feeding sites along the roads might be more relevant factors than those included in the global friction surface. Therefore, further work is needed to build friction surfaces better suited to the specific constrains of animal movement in this environment.

The movement survey database also included volumes of the livestock trade flows between locations. However, these quantitative data were not correctly predicted by any of the models. This failure might be related to two non-exclusive factors. First, when a link was established, the volume of traded animals did not vary much, and not proportionally to the deficit in demand or to the distance. Second, there might be noise in the data related to inaccurate replies during the interviews with field veterinary officers. However, considering the outer trade data (international movements), estimates from this survey closely matched importation estimates from the Senegalese Veterinary Services for the *Tabaski* period. Because these data were of crucial importance to stabilize sheep price (and thus prevent social troubles), all efforts were made for an accurate monitoring of sheep importation. Therefore, this good match provided a partial validation for the quality of data produced by this survey, as well as the good predictive power obtained with the presence/absence models.

An important question underlying these analyses is the role that animal mobility might play in the spread of animal and zoonotic diseases in the region. For example, animal movements may contribute to the spread of Rift Valley fever (RVF) in the Sahelian region of Mauritania, and from Mauritania to Senegal [37,38]. The network structure predicted by this model may provide input for an epidemiological model of RVF or other important diseases affecting the region, such as Peste des petits ruminants (PPR) [6].

Gravity models are an important method in economic analyses, used mainly to predict bilateral flow of population and goods between two distant locations [39]. They were recently adapted to describe the spread of biological agents [33,40–42]. To our knowledge, this study is a first attempt to predict livestock mobility patterns. Besides obvious applications in the field of pastoral economics, it opens new perspectives for predicting the transmission of pathogens such as PPR or RVF viruses in animal meta-populations, or extending existing models of post-vaccination immunity persistence at the population level [43]. Also, similar models might be used at the regional level–e.g, between Maghreb or Sahel countries, or between Sahel and Maghreb regions, etc.–to validate and compliment (un)available information on transboundary animal movements.

## Supporting information

S1 File Small ruminant, cattle and camel trading networks and related centrality measures for Mauritania in 2014. Node size show the importance of the measured centrality values. in and out-weight measures were scaled on the total volume of traded livestock; eigenvector centrality (centrality measure) were scored from 0 to 1, betweenness was considered for the fraction of paths passing through the node.
(DOCX)
