# Supplementary material for: Predictive gravity models of livestock mobility in Mauritania: The effects of supply, demand and cultural factors
Source: PLoS One. 2018 Jul 18;13(7):e0199547. doi: 10.1371/journal.pone.0199547 (PMC6051598; doi:10.1371/journal.pone.0199547)
Supplement: S1 File — Node size show the importance of the measured centrality values. in and out-weight measures were scaled on the total volume of traded livestock; eigenvector centrality (centrality measure) were scored from 0 to 1, betweenness was considered for the fraction of paths passing through the node. (DOCX) [file pone.0199547.s001.docx]

**
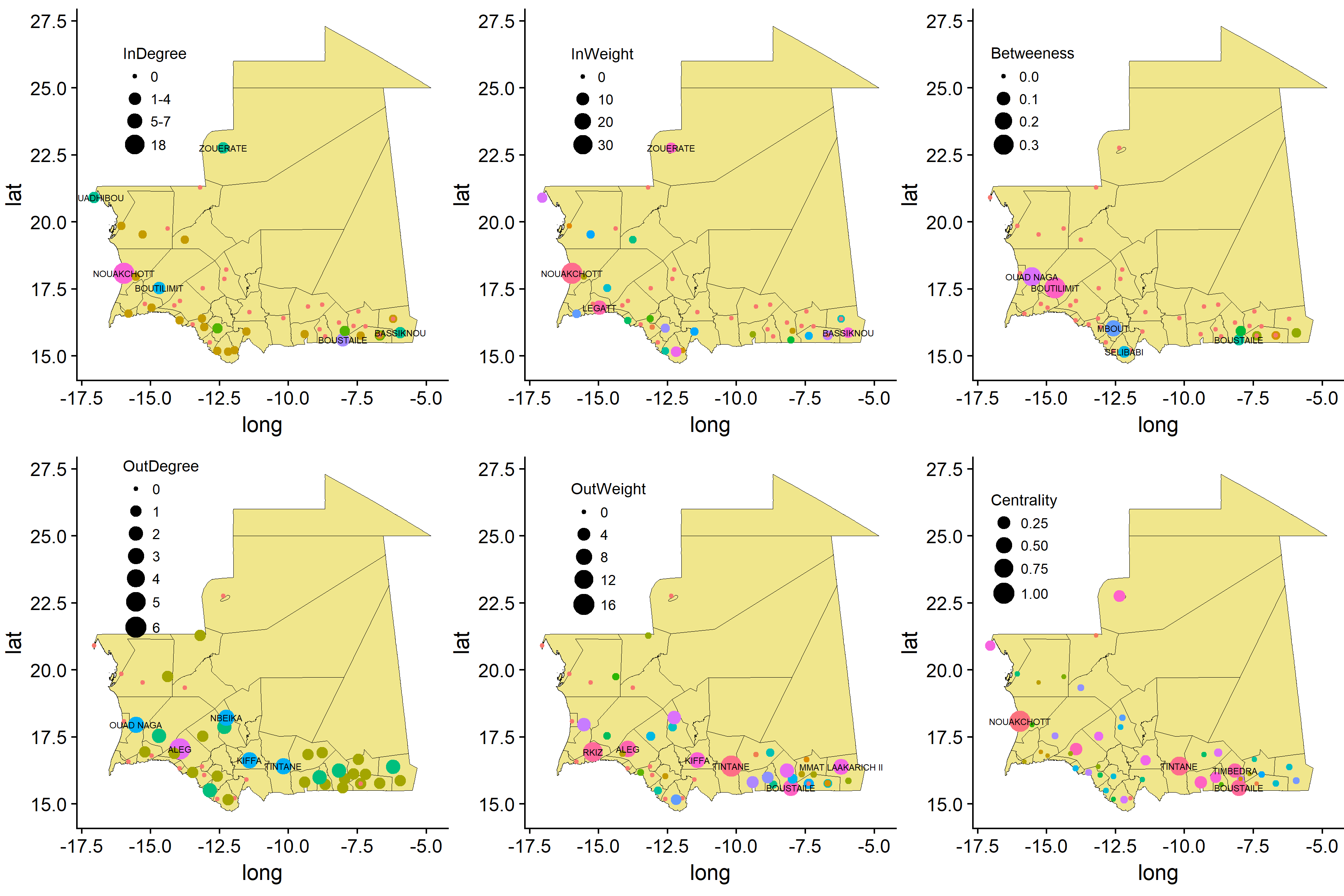
**

**SI Figure 1. Small ruminant trading network and related centrality measures for Mauritania in 2014.** Node size show the importance of the measured centrality values. in and out-weight measures were scaled on the total volume of traded livestock; eigenvector centrality (centrality measure) were scored from 0 to 1, betweenness was considered for the fraction of paths passing through the node.

**
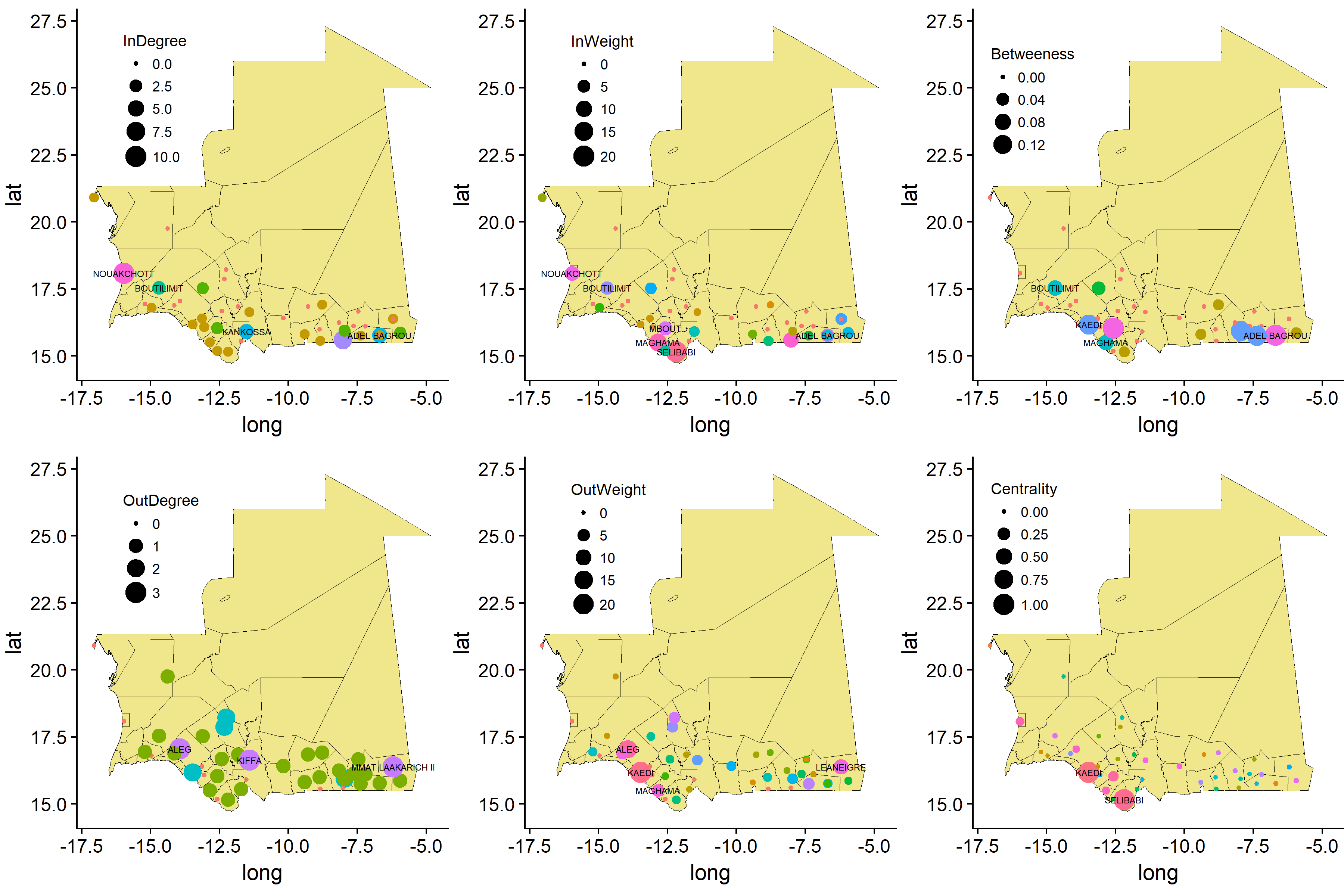
SI Figure 2. Cattle trading network and related centrality measures for Mauritania in 2014.** Legend were kept as in SI Figure 1.

**
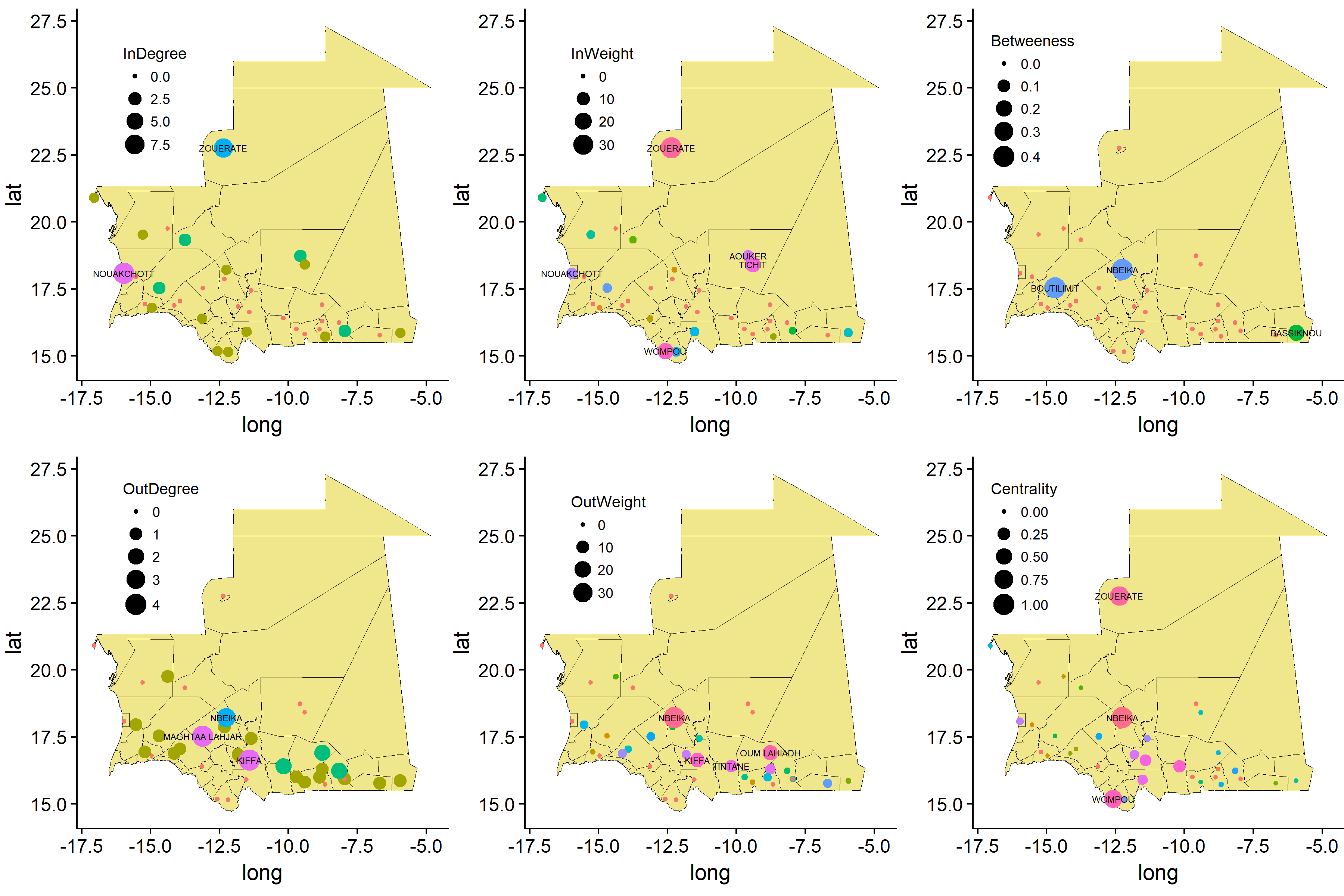
SI Figure 3. Camel trading network and related centrality measures for Mauritania in 2014.** Legend were kept as in SI Figure 1.
